# Harnessing phages in the age of antibiotic resistance: immunological perspectives

**DOI:** 10.3389/fimmu.2026.1866625

**Published:** 2026-07-30

**Authors:** Tasnime A. Abdo Ahmad, Dina Kabbara, Zahraa Shokor, Esber S. Saba

**Affiliations:** 1 Department of Experimental Pathology, Immunology and Microbiology, Faculty of Medicine, American University of Beirut, Beirut, Lebanon; 2 Center of Infectious Diseases Research, Faculty of Medicine, American University of Beirut, Beirut, Lebanon

**Keywords:** antimicrobial resistance, immunogenicity, immunomodulation, innate and adaptive immunities, neutralizing antibodies, phage engineering, phage therapy, phages

## Abstract

The rapid rise of antimicrobial resistance (AMR) has renewed interest in bacteriophages as precision antibacterial agents that can selectively target pathogenic bacteria while limiting disruption of commensal microbiota. However, therapeutic phages are not immunologically inert. Phage particles, phage-derived nucleic acids, bacterial lysis products, and manufacturing impurities can interact with innate and adaptive immune pathways, influencing phage pharmacokinetics, tissue persistence, inflammatory responses, and treatment durability. Innate mechanisms such as complement activation, phagocyte uptake, pattern-recognition receptor signaling, and neutrophil-mediated antibacterial responses may either restrict phage bioavailability or support bacterial clearance. Adaptive immune responses, particularly anti-phage antibodies and serum neutralization, may become relevant during repeated or systemic administration, although available clinical evidence indicates that antibody development does not uniformly predict treatment failure. This review integrates mechanistic, translational, and clinical evidence on phage-immune interactions, distinguishing direct immune recognition of phage components from indirect immune activation mediated by bacteria and bacterial products. We also discuss immune-aware strategies, including phage selection, formulation, route optimization, product-quality control, and immune monitoring, to improve the development of phage therapies for multidrug-resistant bacterial infections.

## 1 Introduction

Phages are viruses that infect and replicate within bacterial hosts. With an estimated global population exceeding 10³¹ particles, they are the most abundant biological entities on Earth and shape microbial ecosystems across soil, oceans, and the human microbiome (1). Their diversity in structure and genome organization underlies their central roles in regulating bacterial communities and promoting horizontal gene transfer (2). Long-term coevolution has driven bacterial defenses such as CRISPR-Cas systems (3) and receptor modification or alterations, with phages evolving counter-strategies including anti-CRISPR proteins (4, 5). These interactions not only shape microbial diversity but also increasingly appear relevant to host immunity. For example, phage-mediated bacterial lysis releases immunostimulatory bacterial products, including lipopolysaccharide, peptidoglycan fragments, and bacterial DNA, which can influence innate immune signaling and inflammatory responses (6). Importantly, the remarkable specificity with which phages infect bacterial hosts, together with their ability to replicate at sites of bacterial infection, has attracted growing interest in their therapeutic potential.

Interest in phage therapy dates to the early 20^th^ century, but the accelerating crisis of antimicrobial resistance (AMR) has renewed global attention to phages as targeted antibacterial agents (7). Unlike broad-spectrum antibiotics, phages can eradicate pathogens while sparing commensals because of their high host specificity, potentially preserving microbiome stability and reducing complications such as antibiotic-associated colitis (8). Recent technological advances, including high-throughput sequencing, metagenomic mining, and synthetic biology, have also transformed discovery and development pipelines, enabling rapid isolation, genomic characterization, rational selection, and personalized or fixed cocktail-based approaches (9, 10). Alongside these developments, early-phase clinical studies and evolving regulatory frameworks support the feasibility and safety of phage therapy across multiple settings (11).

Beyond their antibacterial activity, phages interact with the mammalian immune system in ways that may influence therapeutic success. Contemporary work supports a “tri-kingdom” view in which bacteria, phages, and host immune cells form interdependent networks shaping infection control and inflammatory outcomes (6). Because humans are continuously exposed to phages across mucosal sites, natural (baseline) immune history can shape therapeutic pharmacokinetics and durability (12). During therapeutic administration, innate immune recognition and clearance can limit bioavailability, while adaptive responses may reduce persistence during repeated or systemic dosing (13). Conversely, phages may also contribute to beneficial immunomodulation in select models, including attenuation of excessive inflammation, and phage-based platforms are being explored in non-infectious contexts such as cancer immunotherapy (14–16).

**Figure 1:**
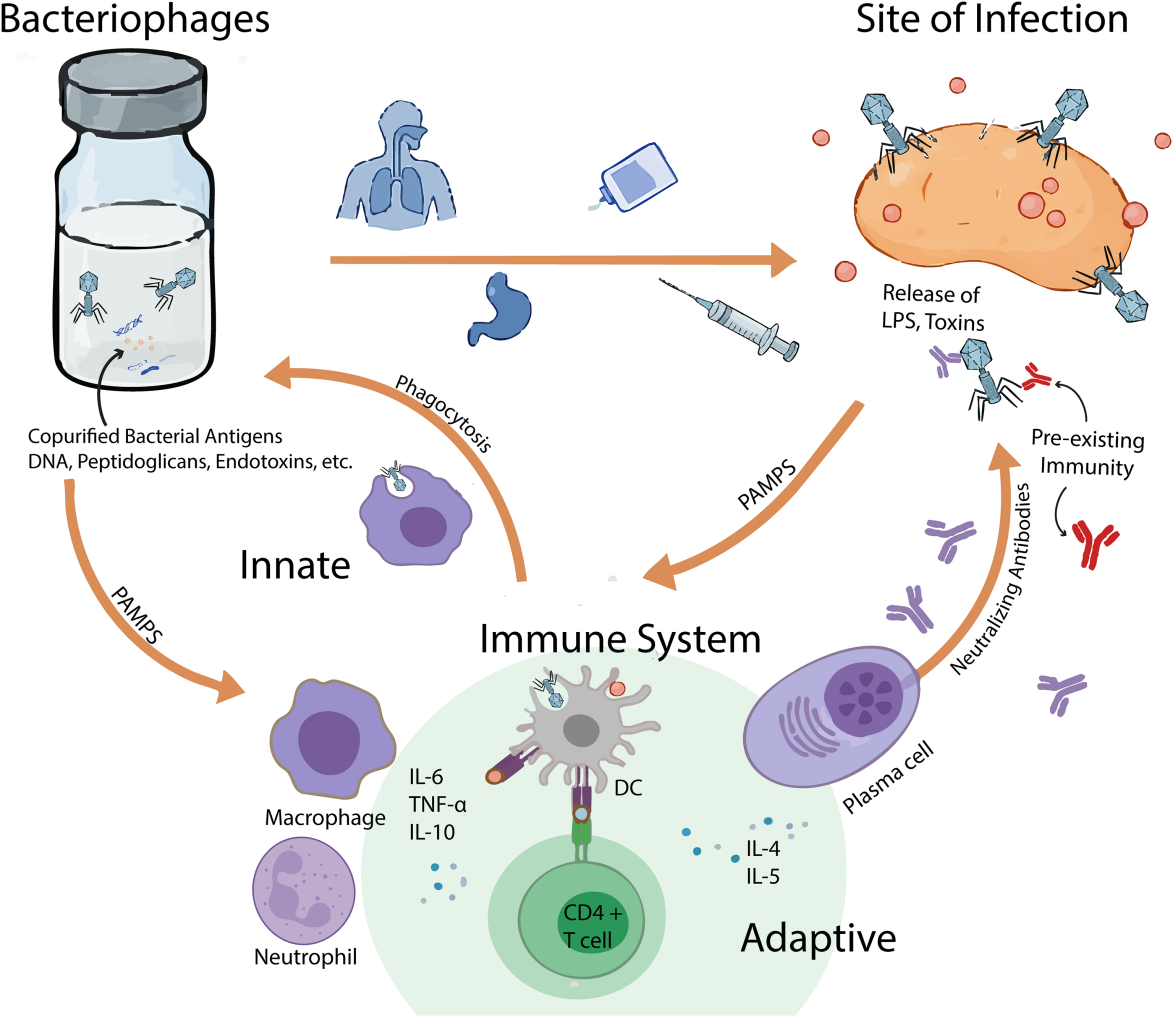
Overview of phage-host immune interactions. At infection sites, phage-mediated lysis releases PAMPs (e.g., LPS, peptidoglycan, bacterial DNA) that activate innate immunity and antigen presentation. Adaptive responses generate antibodies, including phage-neutralizing antibodies, and pre-existing immunity may accelerate phage neutralization and clearance; copurified bacterial products can further contribute to innate sensing.

In this review, we synthesize current evidence on interactions between phages and the mammalian immune system across (i) endogenous phages within the virome/phageome, (ii) exogenous phages administered therapeutically, and (iii) engineered or formulated phage-based products. We distinguish direct immune sensing of phage components from indirect immune activation mediated through bacteria. We examine innate mechanisms that shape early phage clearance and inflammatory outputs, followed by adaptive immune responses that influence durability under repeated dosing. Finally, we integrate the emerging clinical observations to identify determinants of immunogenicity and discuss immune-aware strategies to improve therapeutic efficacy in the AMR era. A central challenge is disentangling immune responses to virions from responses driven by bacterial lysis products and manufacturing impurities (**Figure 1**).

## 2 Immunologically relevant features of phage biology

A clear understanding of phage biology is essential for interpreting how phages interact with mammalian immunity and why immune effects can vary across preparations and clinical contexts. Phage taxonomy has recently undergone major revision: the International Committee on Taxonomy of Viruses ratified changes that replaced several historically morphology-based tailed-phage families (e.g., *Myoviridae*, *Siphoviridae*, *Podoviridae*) and the order *Caudovirales* with a genome-informed framework centered on the class *Caudoviricetes* for tailed dsDNA bacterial and archaeal viruses (17). From an immunology standpoint, this is important because many immune-relevant inferences such as complement sensitivity, antibody targeting patterns, and biodistribution have traditionally been discussed using morphotype language (contractile-tail “myophages,” long non-contractile “siphophages,” short-tail “podophages”). Even if formal taxonomy is no longer morphology-based, morphotype-linked behaviors may remain mechanistically relevant, and several recent studies still compare immune outcomes across morphotypes or taxa that correlate with morphotype (18). In practice, therapeutic development continues to focus largely on tailed dsDNA phages because they are often straightforward to propagate and can be strongly lytic; however, RNA phages and small ssDNA phages remain immunologically informative models for how genome chemistry and cellular compartmentalization influence host sensing (e.g., endosomal RNA sensors versus DNA sensors) (6).

Replication strategy is a central biological determinant of immune engagement because it shapes both bacterial clearance and the inflammatory context in which phages are encountered. During the lytic cycle, phages bind specific bacterial receptors, inject their genome, and co-opt host machinery to generate progeny. Bacterial lysis is mediated by phage-encoded holins and endolysins, resulting in the release of newly formed virions. Because strictly lytic phages efficiently kill their bacterial hosts without establishing lysogeny, they are generally considered the primary candidates for therapeutic applications (8). Yet lysis also releases immunostimulatory bacterial products (e.g., LPS, peptidoglycan fragments, DNA, and, depending on the organism, toxins), creating a recurring interpretive challenge in phage immunology: distinguishing immune responses driven by phage particles themselves from responses to bacterial debris generated during manufacturing or released during bacterial killing *in vivo* (19). In the lysogenic cycle, phage genomes integrate into the bacterial chromosome as prophages and replicate with the host until induction triggers re-entry into lytic growth. Lysogeny contributes to long-term persistence in bacterial populations and promotes horizontal gene transfer, including dissemination of virulence and antibiotic resistance determinants; moreover, prophage carriage can alter bacterial phenotypes (e.g., immune evasion, toxin production, biofilm properties), indirectly shifting host immune responses and confounding attribution of “phage effects” (20). A third phage-host interaction state, pseudolysogeny, can occur when phage genomes persist in a non-replicative, non-integrated state under nutrient-poor or stressful conditions, allowing survival until bacterial physiology improves. Beyond infectious phages, non-infectious phage-derived virus-like particles are increasingly explored as immunotherapy and vaccine platforms because of their stability and intrinsic immunogenicity (21, 22).

Host range and *in vivo* amplification link core molecular biology directly to immunology because they determine where phages replicate, the extent of phage and bacteria-derived immunological stimuli generated during treatment, and how rapidly immune pathways may be engaged (23, 24). Phage-bacterium specificity is largely governed by compatibility between receptor-binding proteins (RBPs) and bacterial surface structures such as lipopolysaccharides, teichoic acids, outer membrane proteins, or capsular polysaccharides. Even minor mutations in RBPs can shift host range by changing binding affinity or enabling recognition of new receptors (25). After adsorption, productive infection requires phages to overcome bacterial defenses, including restriction-modification systems, abortive infection pathways, and CRISPR-Cas immunity, while phages counter through strategies such as DNA modification, anti-CRISPR proteins, or enzymes that degrade structural defenses (5, 26). These determinants influence therapeutic design: high specificity enables precision targeting of pathogens but requires careful selection and characterization and, in some cases, engineering or modular design approaches to broaden host coverage and improve flexibility (27).

Phage structural features further shape immunogenicity and clearance because phage particles are nanoscale, multimeric protein assemblies enclosing nucleic acid, with exposed capsid and tail proteins serving as substrates for B-cell recognition and as potential binding sites for complement and opsonins (12). Recent work illustrates concrete structure-linked immune outcomes: in a serum-based system focused on *Pseudomonas* phages, active complement hindered adsorption of certain myophages, and purified C1q bound phage particles directly (18). In a murine model using a mixed phage cocktail, stronger neutralizing antibody responses were observed against a myophage than against siphophages, and the resulting immune state reduced efficacy upon reuse (28). At mucosal surfaces, the Bacteriophage Adherence to Mucus (BAM) model proposes that some phages adhere to mucins via Ig-like capsid domains, enriching phages at epithelial interfaces and potentially providing a non-host-derived antimicrobial barrier; immunologically, such localization may influence both exposure at immune-rich boundaries and indirect modulation of inflammation through effects on colonization resistance (29).

Finally, “phage purity” is itself an immune-relevant biological variable: what is labeled a “purified phage” preparation can differ markedly in residual bacterial material (e.g., endotoxin, membrane fragments, proteins, nucleic acids), excipients, and manufacturing by-products, each capable of driving innate activation and confounding interpretation of phage-specific effects (19). For this reason, mechanistic conclusions about direct host sensing of phages depend on careful definition of phage type (including morphotype/genome class and life cycle), host range/amplification potential, and preparation quality/composition (30, 31).

## 3 The human phageome and pre-existing immunity

Humans are continuously exposed to phages through multiple routes, including the gastrointestinal tract, oral ingestion, inhalation, and contact with skin and mucosal surfaces (32, 33). Consequently, recurrent exposure to phage particles is likely a normal feature of human physiology. This exposure is facilitated by the high abundance of phages in the intestinal ecosystem, where virus-like particle concentrations are commonly reported in the range of ~10^8^-10^9^ particles per gram of fecal material or gut content, with phages comprising the majority of viral particles (34). Persistent phage-mucus-epithelium interactions at densely colonized mucosal sites (29), together with repeated environmental exposure through water, food, and built environments (35), suggest that the gut may serve as a major interface for phage-host interactions and that individual immune exposure histories are likely to vary substantially (36).

Consistent with this, the human phageome is niche-structured and often person-specific, tracking local bacterial ecology and physicochemical constraints such as mucus, oxygen tension, pH, flow, and immune accessibility (37). Deep longitudinal gut virome studies have shown that individual viral consortia can persist for months to years (38). In addition, large-scale cataloging efforts, including analyses of tens of thousands of gut metagenomes yielding on the order of 10^5^ non-redundant viral genomes >10 kb, highlight both the remarkable diversity of the human phageome and the extent of incomplete sampling (39). Recurrently observed gut components (e.g., crAss-like and Microviridae-like genomes), together with site-specific patterns outside the gut (saliva, skin, and airway), may contribute to local immune priming (40, 41).

In the phage-therapy literature, “pre-existing immunity” most often refers narrowly to measurable baseline humoral activity against phage virions (binding antibodies by ELISA/serology), yet these readouts are not interchangeable because binding can occur without neutralization, and neutralization may require antibodies targeting infectivity-critical structures (tail/baseplate/RBPs) rather than internal or non-neutralizing epitopes (42, 43).

A broader and clinically important definition also includes pre-existing innate and Fc-mediated clearance pathways that limit systemic bioavailability even when neutralizing titers are low, including complement inactivation, opsonization and phagocytic uptake, and rapid sequestration by the liver/spleen mononuclear phagocyte system (44–46). Compartmentalization is central because baseline immunity can differ sharply between mucosal secretions (where sIgA and local innate factors dominate) and blood (where IgG/IgM and complement predominate) (47), meaning the exposure route (oral/topical/inhaled/injected) determines which immune compartment is engaged and therefore what “pre-existing” means for a given therapeutic strategy (42, 48, 49).

Direct evidence in healthy humans supports both prevalence and heterogeneity of baseline anti-phage activity: classic data with T4 coliphage reported high rates of baseline neutralization in apparently unexposed volunteers and strong natural IgG recognition focused on major capsid determinants consistent with cross-reactivity among T4-like phages (50), whereas other studies testing healthy donor collections against therapeutically relevant *Pseudomonas* PB1-like and *staphylococcal Herelleviridae* phages found markedly lower and phage-dependent baseline neutralization frequencies and generally moderate potency, with many donors neutralizing one close relative but not another (51, 52).

Mechanistically, several layers link the phageome to baseline immunity: mucus association (e.g., BAM-like retention) can concentrate phages at epithelial surfaces and increase immune sampling (29); epithelial transcytosis can provide low-fraction but potentially meaningful systemic exposure under chronic high-burden conditions and may be amplified by inflammation-associated barrier defects (53); and once systemic, phage fate is shaped by the combined action of phagocytes, antibodies, and complement, with repeated dosing *in vivo* commonly accelerating clearance and increasing neutralizing activity (44).

Structural context matters because immunogenicity is not uniform across virion proteins (immunodominance can fall on major capsid or exposed accessory proteins, and recombinant proteins may elicit binding without neutralization), so baseline serology alone is an imperfect proxy for functional inactivation (54). In addition to structural context, anatomical context may also influence phage immunogenicity and clearance, as different tissues possess distinct immune environments that can shape phage persistence and host responses (55).

Clinically, the impact of baseline immunity depends on route, duration, and whether efficacy requires sustained systemic exposure versus local amplification at the infection site: oral delivery often produces minimal systemic exposure and weak systemic antibody induction in short courses (49, 56), whereas inhaled/nebulized and especially IV regimens can induce neutralization on clinically relevant timescales (57); importantly, cohort studies and case reports indicate that emergence of serum antiphage activity does not function as a simple “failure switch,” with outcomes sometimes independent of antibody magnitude in indications dominated by local pharmacodynamics (57).

Collectively, these observations support an expanded, evidence-based view in which pre-existing anti-phage immunity is common but unevenly distributed, phage-specific rather than a single host trait, and strongly compartmentalized. These properties argue for immune-aware trial design including phage-by-phage functional neutralization screening (not only binding assays), antigenic rotation/substitution strategies, and route-targeted delivery to minimize systemic neutralization pressure when systemic bioavailability is not required.

## 4 Innate immune recognition of phages

The innate immune system is the first biological barrier encountered by both endogenous phages at mucosal surfaces and exogenous phages administered therapeutically (topical, mucosal, inhaled, or systemic), and it is a decisive determinant of phage pharmacokinetics, tissue persistence, and antibacterial efficacy (58). Although phage-bacterium interactions have historically dominated the field, it is now clear that phage-immune interactions critically shape therapeutic success. Following intravenous (IV) or mucosal administration, phages rapidly encounter professional phagocytes, including macrophages, dendritic cells (DCs), neutrophils, and monocytes (59). Several partially redundant innate mechanisms can act in parallel, including pattern-recognition receptor (PRR) signaling, complement-mediated inhibition, phagocyte uptake/processing, and (in some contexts) neutrophil effector programs such as NETosis (31). Despite their nanoscale size, phages are efficiently internalized through receptor-mediated endocytosis and phagocytosis (60), and a mechanistic overview of these pathways has been synthesized in recent reviews and conceptual maps (21).

A central question for PRR-mediated recognition is where and how phage nucleic acids become accessible to host sensors. Because phages do not replicate in mammalian cells, nucleic-acid sensing likely requires uptake of intact particles followed by endosomal processing (61), uptake of degraded particles, or exposure to nucleic acids released during bacterial lysis at sites where host sensors are present (62). Endosomal sensing pathways provide one plausible route: TLR7 and TLR9 can detect viral RNA and unmethylated CpG-rich DNA, respectively, via MyD88-dependent signaling programs that induce type I interferons and inflammatory cytokines (63). Cytosolic surveillance adds another layer, in which cGAS binding to double-stranded DNA can activate STING-TBK1-IRF3 signaling (63–65), and AIM2-like receptors can support inflammasome-mediated DNA sensing with downstream IL-1β production (66). However, emerging data emphasize that “phage” is not a single immunological stimulus: PRR engagement can vary by phage genome type and context, and recognition may be partial or non-canonical (67). In a chicken *Salmonella* phage-therapy model, phage DNA increased TLR9-associated signals and IFNβ elevation was observed, yet canonical cGAS-STING antiviral signaling was interrupted upstream of IRF3 phosphorylation due to failure of RNA polymerase III-dependent dsRNA generation from phage DNA (68). Complementary *in vivo* observations similarly support measurable innate sensing without overt inflammatory pathology in some settings (69) and cytokine profiling under oral high-dose administration has shown mixed outputs (e.g., TNF-α and IL-6 alongside regulatory mediators such as IL-10 and IL-4), consistent with context-dependent immune activation that can preserve homeostasis relative to antibiotic-treated controls (68, 69).

Beyond PRRs, complement can function as an antiviral-like neutralizer of phage infectivity and a key determinant of whether *in vitro* activity translates to *in vivo* performance. In a detailed study of human serum effects on phage infectivity, complement activation hampered phage adsorption to bacterial hosts. Mechanistically, purified C1q bound directly to phage particles in a concentration-dependent manner, and blocking C1q rescued phage binding and function in the tested system. Inhibition was prominent for myophages in that panel, motivating morphotype-aware phage selection and the concept of serum-inclusive prescreening for therapeutic candidates (18). These findings reinforce a practical translational point: phage performance in buffered culture conditions may not predict performance in complement- and opsonin-rich environments, highlighting the need for physiologically relevant assay conditions during development (19).

Phagocyte uptake is both a recognition mechanism and a pharmacokinetic bottleneck, shaping biodistribution and clearance kinetics after systemic or inhaled delivery (58). A well-characterized molecular example is phage T4, whose Highly Immunogenic Outer Capsid (Hoc) protein contains a lysine-glycine-aspartic acid (KGD) motif that mimics the mammalian RGD integrin-binding sequence, enabling interaction with β3 integrins and promoting uptake by phagocytes (70). Once internalized, phages traffic through endosomal-lysosomal pathways where they are degraded and/or processed for antigen presentation (60). In murine models of *Pseudomonas aeruginosa* (PSA) pneumonia, alveolar macrophages rapidly phagocytosed inhaled phages, sharply reducing pulmonary phage titers; macrophage depletion prolonged phage persistence and significantly improved bacterial clearance, demonstrating that innate clearance can act as a therapeutic bottleneck rather than a passive variable (36, 71). Broader pharmacokinetic analyses similarly support that innate immune elimination strongly shapes systemic exposure and therapeutic durability (72). Controlled *in vivo* pharmacokinetic work also illustrates rapid loss of functional plaque-forming particles from blood with accumulation in filtration organs such as the spleen, and discrepancies between plaque assay and qPCR suggest substantial loss of functional phage relative to detectable phage material (73). A perspective review synthesizes multiple datasets and reports that IV administration of diverse phages in mice and humans can yield ≥99% removal from blood within 6 hours with primary accumulation in liver and spleen, highlighting roles for Kupffer cells and splenic macrophages and emphasizing opsonization as a bridge between innate and adaptive clearance (55).

Neutrophils are central to bacterial infection outcomes, making it clinically important to distinguish whether phages directly activate neutrophils in harmful ways versus whether neutrophils contribute indirectly to therapeutic success. In an *in vitro* human neutrophil study using a purified therapeutic candidate *Pseudomonas* phage (PAK_P1), high phage concentrations induced a modest (~2-fold) IL-8 increase but did not trigger apoptosis/necrosis, oxidative burst, or NET formation; moreover, phage exposure did not amplify NETosis induced by either strong or weak stimuli, suggesting that at least some purified lytic phages are unlikely to directly drive NET-mediated tissue damage (71). At the same time, innate immunity does not solely restrict phage therapy, it can potentiate it. In the *P. aeruginosa* pneumonia model, neutrophils were required for therapeutic success, and neutrophil depletion abolished bacterial control despite phage administration, supporting immunophage synergy and cooperative killing of both phage-sensitive and phage-resistant bacteria (71). Reviews further describe functional amplification of antibacterial responses in inflamed tissues through phage-neutrophil interactions (74). Across these findings, a critical interpretive caveat is that innate signatures are not purely “host versus phage”: they vary with route, dose, infection state (which provides bacterial replication niches and alters immune tone), and product impurities such as endotoxin, which can strongly confound *in vitro* and *in vivo* readouts (75). Taken together, innate immunity acts as a dual regulator of phage therapy: PRR signaling, complement, and phagocytic clearance can limit persistence and reduce effective exposure, whereas neutrophil cooperation and context-dependent activation can enhance bacterial clearance and shape inflammatory tone (30).

## 5 Adaptive immune response to phages

Following innate sensing and processing, phages can elicit adaptive immune responses that influence long-term therapeutic efficacy. Adaptive immunity to phages encompasses antigen presentation with T cell help, class-switched antibody production (neutralizing and non-neutralizing), and the establishment of immunological memory that can reshape subsequent exposures (55). Across models, phage structural proteins are prominent antigenic substrates, and antibody-mediated immunity appears to be the dominant adaptive arm in most therapeutic contexts (28). Mechanistically, phage-specific antibodies can reduce antibacterial activity by directly neutralizing phage infectivity (e.g., blocking bacterial adsorption sites) and can accelerate clearance through opsonization, complement activation, and Fc receptor-mediated uptake by phagocytes (55). A recent synthesis on phage-specific antibodies emphasizes these dual roles while also underscoring that clinical evidence remains inconsistent and that no consensus exists on when antibody responses reliably predict therapeutic failure (12).

Evidence for adaptive memory effects comes from a murine model of intestinal decolonization using a five-phage cocktail targeting vancomycin-resistant *Enterococcus*. After two treatment courses separated by a memory interval, phage exposure induced phage-specific IgG against all phages and increased memory/plasma B cell phenotypes, with negligible T cell responses in the measured settings (28). Importantly, pre-exposure reduced phage persistence (including faster splenic clearance) and reduced efficacy when the same cocktail was reused, providing direct evidence for a causal pathway from anti-phage immune memory to diminished therapeutic effect (28). Notably, antibody magnitude differed between phages within the same cocktail: stronger neutralizing responses were observed against a myophage than against siphophages, reinforcing the broader concern that immunogenicity can vary with phage type and structural class. These dynamics are consistent with the view that repeated exposure enhances class-switched neutralizing responses and accelerates clearance upon re-administration, such that adaptive immunity can progressively amplify elimination over time (28).

A synthesis review notes stronger antibody induction after intraperitoneal or IV exposure than after oral or topical routes, while stressing that phage identity, dosing schedule, and host immune state can modify these tendencies (30). Importantly, baseline neutralizing antibodies can exist in individuals without prior phage therapy supporting the rationale for pre-treatment serum screening in some development pipelines, particularly for products intended for systemic administration or repeated use (12). At the same time, antibody formation does not uniformly predict failure: effective bacterial clearance can occur despite detectable anti-phage IgG, plausibly reflecting factors such as high local phage replication at infection sites, spatial compartmentalization that limits antibody access, and synergistic phage-immune antibacterial effects (12). Finally, phage immunogenicity is not only a barrier to therapy but also an opportunity: engineered phage and virus-like particle platforms (including T4-derived systems) are being developed for antigen display and vaccine or immunotherapy applications, leveraging robust humoral, cellular, and mucosal immune responses without pathogenic risk (16). Collectively, adaptive immunity to phages functions as a double-edged regulator underscoring the importance of optimizing phage identity, route, and dosing schedules for durable and personalized phage-based approaches.

To provide an overview of the concepts discussed above, **Table 1** summarizes the major innate and adaptive immune mechanisms involved in phage recognition and clearance, as well as their potential consequences for phage therapy.

**Table 1:** Innate and adaptive immune mechanisms involved in phage recognition and clearance.

Immune mechanism	Main trigger	Cells/pathways involved	Possible effect on therapy	Translational implication	Study
PRR sensing	Phage DNA/RNA, capsid proteins, phage-associated PAMPs	TLR3, TLR7/8, TLR9, cGAS-STING, dendritic cells, macrophages	Innate immune activation and cytokine production	May influence immunogenicity and route-specific responses	Górski et al., 2017; Van Belleghem et al., 2018; Roach & Debarbieux, 2017 (28, 48, 76, 77)
Complement activation	Interaction of circulating phages with serum complement	Classical, alternative, lectin pathways	Opsonization and phage clearance	Can reduce systemic bioavailability after IV administration	Hodyra-Stefaniak et al., 2015; Van Belleghem et al., 2018 (44, 48)
Phagocyte uptake	Recognition of phages by innate immune cells	Macrophages, monocytes, Kupffer cells, splenic phagocytes	Rapid removal from circulation	Important determinant of pharmacokinetics and dosing	Merril et al., 1996; Hodyra-Stefaniak et al., 2015 (44, 78)
Antibody binding	Repeated/prolonged phage exposure	B cells, plasma cells, IgM, IgG, IgA	Reduced persistence and altered biodistribution	Important for chronic therapy and repeat dosing	Żaczek et al., 2016; Łusiak-Szelachowska et al., 2020 (51, 79)
Serum neutralization	Development of neutralizing antibodies	Anti-phage IgG/IgM	Direct loss of infectivity	May contribute to treatment failure during long-term IV therapy	Dedrick et al., 2021; Dedrick et al., 2023 (80, 81)
Fc-mediated clearance	Antibody-coated phage particles	Fcγ receptor-bearing macrophages and monocytes	Accelerated clearance despite preserved infectivity	May reduce circulating phage concentrations	Dąbrowska 2019; Van Belleghem et al., 2018 (48, 82)
Inflammatory response to bacterial lysis products	Release of endotoxin, peptidoglycan, bacterial debris after lysis	TNF-α, IL-1β, IL-6 pathways	Transient inflammatory reactions	May be misinterpreted as treatment failure or infection worsening	Dufour et al., 2017; Van Belleghem et al., 2018 (48, 83)
Impurity/endotoxin-driven inflammation	Endotoxin, bacterial DNA, host-cell proteins in preparations	TLR4 and innate inflammatory pathways	Fever, infusion reactions, cytokine release	Highlights importance of purification and GMP production	Aslam et al., 2020; Petrovic Fabijan et al., 2020; Pirnay et al., 2024 (84–86)
Adaptive cellular immune response	Presentation of phage-derived antigens	CD4^+^ T cells, Tfh cells, APCs	Supports antibody production and immune memory	Relevant for prolonged therapy and repeated courses	Démoulins et al., 2025; Van Belleghem et al., 2018 (48, 87)
Mucosal immune response	Exposure at respiratory, GI, or urinary mucosa	Secretory IgA, mucosal APCs	Local neutralization of phages	Important for inhaled, oral, intranasal, and intravesical therapy	Letkiewicz et al., 2021; Bernabéu-Gimeno et al., 2024 (88, 89)

APCs, antigen-presenting cells; cGAS, cyclic GMP–AMP synthase; GI, gastrointestinal; IgA, immunoglobulin A; IgG, immunoglobulin G; IgM, immunoglobulin M; IL, interleukin; IV, intravenous; PAMPs, pathogen-associated molecular patterns; PRR, pattern-recognition receptor; STING, stimulator of interferon genes; Tfh, T follicular helper; TLR, Toll-like receptor; TNF-α, tumor necrosis factor alpha.

## 6 Indirect immune effects mediated through bacteria

Beyond direct interactions with mammalian immune cells, phages exert a second, indirect immunological axis through their effects on bacterial communities and bacterial products (90). First, phage-driven reshaping of the microbiome can influence immune homeostasis by modulating bacterial composition and abundance, thereby altering microbial-derived signals that calibrate mucosal immunity. This may shift tolerogenic vs inflammatory signaling depending on which bacterial taxa expand or shrink (91, 92).

Phage-mediated bacterial lysis releases immunogenic components including endotoxin and bacterial DNA, which can modulate cytokine production and inflammatory signaling; experimental models demonstrate that immune activation varies depending on phage type, bacterial load, and infection context (93, 94). Clinically, this is why endotoxin/impurity control and dosing escalation are emphasized for systemic products.

Prophages embedded in bacterial genomes can encode traits that alter host-microbe interactions, including virulence factors, toxins, superantigens, and immune evasion determinants, thereby directly shaping bacterial immunogenicity and pathogenic potential (95). Consequently, phage-bacteria interactions may either attenuate inflammation or exacerbate inflammatory outcomes when lysis products accumulate. This makes prophage content a safety/selection consideration when choosing bacterial hosts or evaluating lysogeny-related risks (92, 96).

Taken together, these findings indicate that phage therapy outcomes depend not only on direct immune recognition of phage particles but also on context-dependent effects mediated through bacterial ecology, lysis-derived immunostimulants, and prophage-encoded functions. These indirect mechanisms may either attenuate or exacerbate inflammation depending on microbial burden, tissue site, and host immune status.

## 7 Human evidence: What do clinical and real-world data show?

Clinical and translational evidence linking phages to immunity spans (i) a limited number of randomized controlled trials, (ii) early-phase trials primarily assessing safety and feasibility, and (iii) an expanding body of observational cohorts and case reports with heterogeneous methodologies, endpoints, and immune readouts (49, 86, 97). Across these study types, what has been measured most directly includes anti-phage IgG/IgM titers, serum neutralization assays, circulating cytokines, and pharmacokinetic clearance profiles, yet these markers do not yield a single rule that reliably predicts clinical success or failure.

However, systematic immune monitoring has not yet been incorporated into most clinical phage therapy studies, which have historically focused on safety, tolerability, and microbiological or clinical outcomes. Consequently, current data on anti-phage immune responses remain limited and are derived primarily from a relatively small number of studies in which immune endpoints were included as exploratory measures (8, 98, 99).

In the randomized PhagoBurn trial, systemic inflammatory signals were modest and safety acceptable, but efficacy challenges were more strongly linked to phage concentration/product potency and bacterial susceptibility than to immune parameters, and neutralization was not clearly associated with outcomes (49). Prospective and compassionate-use experiences similarly show that anti-phage antibodies and serum neutralization frequently rise during prolonged IV therapy and may coincide with reduced circulating phage activity, but clinical improvement often occurs despite measurable neutralization, especially when phage replication at the infection site is sustained (84, 100). This heterogeneity is evident across indications: in a pulmonary *Mycobacterium abscessus* case treated intravenously with a three-phage cocktail, initial reduction in bacterial burden was followed by diminished efficacy concurrent with robust IgM/IgG neutralization. This supports the view that humoral immunity can limit long-term systemic benefit in some contexts (80), whereas in the 20-patient mycophage compassionate-use series, neutralizing antibodies emerged in a subset and may have contributed to nonresponse in some patients but were not consistently outcome-determining (80).

In cystic fibrosis (CF) and chronic airway infections, inhaled or combined systemic-local administration can generate systemic neutralizing signals (detected 10–42 days post-therapy) without clear safety concerns in that series, reinforcing that route influences immunogenicity but does not straightforwardly map onto failure (89). Large real-world data further support a “modulator” interpretation: in a 100-case multicenter cohort of consecutive personalized phage therapy, serum immune neutralization was observed in 38.5% (5/13) of screened patients, yet overall clinical improvement was reported in 77.2% and bacterial eradication in 61.3%, with antibiotics commonly co-administered and associated with higher eradication odds (86). Clinical experience therefore points to substantial inter-individual variability in host immune responses, in which repeated or systemic therapy often induces phage-specific humoral responses (IgM/IgG) that can bind virions, block adsorption, activate complement, and accelerate clearance. Yet, antibody development does not uniformly predict therapeutic failure because *in situ* phage replication, tissue compartmentalization and limited antibody access to infected niches can preserve efficacy. Successful outcomes have been documented even alongside robust adaptive responses, including a lung transplant recipient with MDR *PSA* infection who developed phage-specific and highly individualized IgG titers and a measurable CD4^+^ T-cell response while still achieving marked clinical improvement and bacterial clearance (101), as well as a patient with disseminated *M. abscessus* treated with engineered mycophages in whom detectable neutralizing antibodies did not prevent sustained microbiological and clinical benefit (42).

Route of administration remains a major determinant of immunogenicity, with oral delivery typically associated with low systemic titers (consistent with mucosal tolerance and limited translocation), systemic routes (IV/intramuscular) more likely to drive IgM/IgG induction via direct exposure to circulating immune cells and lymphoid tissues, and topical/inhalational routes producing intermediate patterns that may vary with local inflammation and dosing frequency (48). Importantly, immune responses are also phage-specific: different phages within the same cocktail can elicit distinct antibody profiles reflecting differences in capsid/tail architecture and exposed epitopes, supporting immune-aware strategies such as phage rotation, selection of antigenically non-cross-reactive phages, rational cocktail design, and formulation approaches (e.g., encapsulation or polymer shielding) to mitigate neutralization during repeated dosing.

Collectively, these findings suggest that anti-phage titers and neutralization assays are best viewed as pharmacodynamic markers of exposure durability whereas in localized, short-course, or strongly site-replicative therapy, determinants such as bacterial susceptibility, delivery route, local phage kinetics, and product potency/quality may be more informative than serum neutralization alone. This perspective increasingly aligned with translational emphasis on standardized characterization, purity/impurity control, and consistency for phage therapy medicinal products.

**Table 2** summarizes key clinical and real-world phage therapy studies that reported immune-related outcomes, including antibody responses, neutralization, cytokine changes, safety findings, and therapeutic outcomes.

**Table 2:** Human clinical evidence on immune responses during phage therapy.

Study	Study type	Infection/condition	Route	Phage product	Immune assay/marker	Main immune finding	Clinical/microbiological outcome	Interpretation/limitations
Bruttin & Brüssow, 2005 (102)	Safety trial	Healthy adult volunteers	Oral	*E. coli* phage T4	Anti-phage antibodies	No detectable antibody induction	Safe; no infection outcome	Low-dose oral exposure may be weakly immunogenic
Schooley et al., 2017 (100)	Compassionate case report	MDR *Acinetobacter baumannii*	IV + intracavitary	Personalized phage cocktails	Safety/inflammatory monitoring	No major immune-mediated toxicity reported	Clinical recovery	Immune monitoring limited
Dedrick et al., 2019 (42)	Case report	Disseminated *Mycobacterium abscessus* subsp. *massiliense*	IV ± topical	Engineered/selected mycophages	Anti-phage antibodies, neutralization	Weak antibody response; no strong neutralization	Clinical improvement	Single case; immunosuppressed host
Ooi et al., 2019 (103)*	Phase I trial	*S. aureus* chronic rhinosinusitis	Intranasal	AB-SA01	Safety labs/adverse events	No systemic inflammatory toxicity	Safe; 2/9 eradicated S. aureus	Limited immune profiling
Petrovic Fabijan et al., 2020 (85)*	Prospective clinical study	Severe *S. aureus* infections	IV	AB-SA01	Safety labs, phage kinetics	No immune-mediated adverse reactions	Safe; clinical improvement in several cases	Mainly safety-focused
Aslam et al., 2020 (84)*	Case series	MDR bacterial infections	IV	Personalized phages	Safety monitoring	No serious immune toxicity reported	7/10 favorable outcomes	Immune assays not uniform
Rubalskii et al., 2020 (104)*	Case series	Cardiothoracic surgery infections	Local/systemic	Personalized phages	Adverse events/safety	No severe immune adverse effects	Target bacteria eradicated in 7/8	Limited mechanistic immune data
Cano et al., 2021 (105)	Case report	*Klebsiella pneumoniae* prosthetic knee infection	Local/intra-articular ± IV	Personalized phages	Anti-phage antibody/neutralization monitoring	No clear antibody-mediated loss of activity	Infection controlled; limb/prosthesis salvaged	Single case
Dedrick et al., 2021 (80)	Case report	Pulmonary *M. abscessus* infection	IV	Three-phage cocktail	IgM, IgG, neutralization	Strong neutralizing antibodies developed	Initial bacterial fall, then rebound	Strong example of antibody-limited efficacy
Letkiewicz et al., 2021 (88)	Observational study	Chronic urinary/urogenital infections	Intravesical ± intravaginal	Therapeutic phages	Serum anti-phage antibodies	Single course weakly immunogenic; second course increased antibodies but remained low	Some favorable responses	Local delivery appears low-immunogenic
Nick et al., 2022 (106)	Translational case study	*M. abscessus* lung infection	IV	Engineered mycophages	Neutralizing antibodies	Antibodies rose to one phage but did not prevent response	Clinical improvement; explanted lung culture-negative	Antibodies not always clinically limiting
Dan et al., 2022/2023 (101)	Immunology case report	MDR *P. aeruginosa* pneumonia	Adjunctive phage therapy	Personalized phages	CD4 T cells, cTfh cells, IgG, neutralization	Cellular and humoral anti-phage responses developed	Clinical success	Single transplant-recipient case
Hahn et al., 2023 (107)	Case series	CF airway infection	Inhaled/nebulized	Personalized phages	Antibody/neutralization monitoring	Anti-phage immunity assessed; limited reported clinical impact	Variable respiratory outcomes	Small CF cohort
Le et al., 2023 (108)	Translational case report/study	Bacterial infection treated with phage	Reported clinical route	Personalized phages	Anti-phage antibodies	Strong serum antibody response did not necessarily impair outcome	Favorable response reported	Details depend on individual case context
Green et al., 2023 (109)	Retrospective expanded-access cohort	Mixed MDR infections	Mixed routes	Customized phages	Neutralization assays in subset	Neutralization monitored in selected cases	Clinical improvement in many cases	Heterogeneous infections and assays
Saima Aslam et al., 2024 (110)	Case series	*P. aeruginosa* LVAD infections	IV	Personalized Phages	Serum neutralizing antibodies	Neutralization developed in some patients	Often ineffective; breakthrough bacteremia	Device biofilm and antibodies may limit efficacy
Pirnay et al., 2024 (86)	Retrospective cohort, 100 case	Personalized therapy for diverse infections	Mixed routes	Magistral/personalized phages	Neutralization testing	Neutralizing antibodies detected in subset	77.2% clinical improvement; 61.3% eradication	Large but heterogeneous cohort
Bernabéu-Gimeno et al., 2024 (89)	CF case series	CF lung infections	Nebulized	Monophage treatments	Serum neutralizing antibodies	Neutralizing antibodies emerged after nebulization	3–6 log bacterial reduction in 2 treatments	Shows inhaled phage can still induce antibodies
Łusiak-Szelachowska et al., 2014 (51)	Observational immune-monitoring study	Patients receiving phage therapy	Oral/local	Therapeutic phages	Anti-phage antibodies	Antibody responses varied	Outcomes not clearly predicted by antibodies	Early clinical immune-monitoring dataset
Żaczek et al., 2016 (79)	Observational study	*S. aureus* infections	Oral/local	MS-1 cocktail	IgG, IgA, IgM, neutralization	Most patients had modest antibody responses	Antibodies did not clearly predict failure	Small heterogeneous cohort
Łusiak-Szelachowska et al., 2020 (111)	Observational cohort	Chronic sinusitis	Local	Therapeutic phages	Serum anti-phage antibodies	No correlation between antibody level and outcome	Similar outcomes in low/high antibody groups	Serum antibodies alone may be insufficient marker
Łusiak-Szelachowska et al., 2022 (112)	Follow-up immune study	Prior phage-treated patients	Prior oral/local therapy	Therapeutic phages	Persistence of anti-phage antibodies	Anti-phage antibodies may persist after therapy	Clinical relevance unclear	Long-term antibody persistence needs more study
Łusiak-Szelachowska et al., 2025 (43)	Observational follow-up study	Patients undergoing phage therapy	Oral/local	Therapeutic phages	Serum anti-phage antibodies	Antibodies appeared in some patients but did not consistently predict outcome	Outcome’s variable	Supports need for standardized immune monitoring

CF, cystic fibrosis; CFU, colony-forming units; MDR, multidrug-resistant; PSA, Pseudomonas aeruginosa; LVAD, left ventricular assist device; IV, intravenous; PEG, polyethylene glycol; PAS, phage adsorption to mucus.

*These studies primarily reported standard clinical safety, tolerability, adverse events, and routine laboratory monitoring during phage therapy and did not perform dedicated immunological or serological assays (e.g., anti-phage antibody quantification, neutralization testing, or cellular immune profiling). Their inclusion provides supportive safety-related observations rather than direct evidence of phage-induced immune responses.

**Figure 2:**
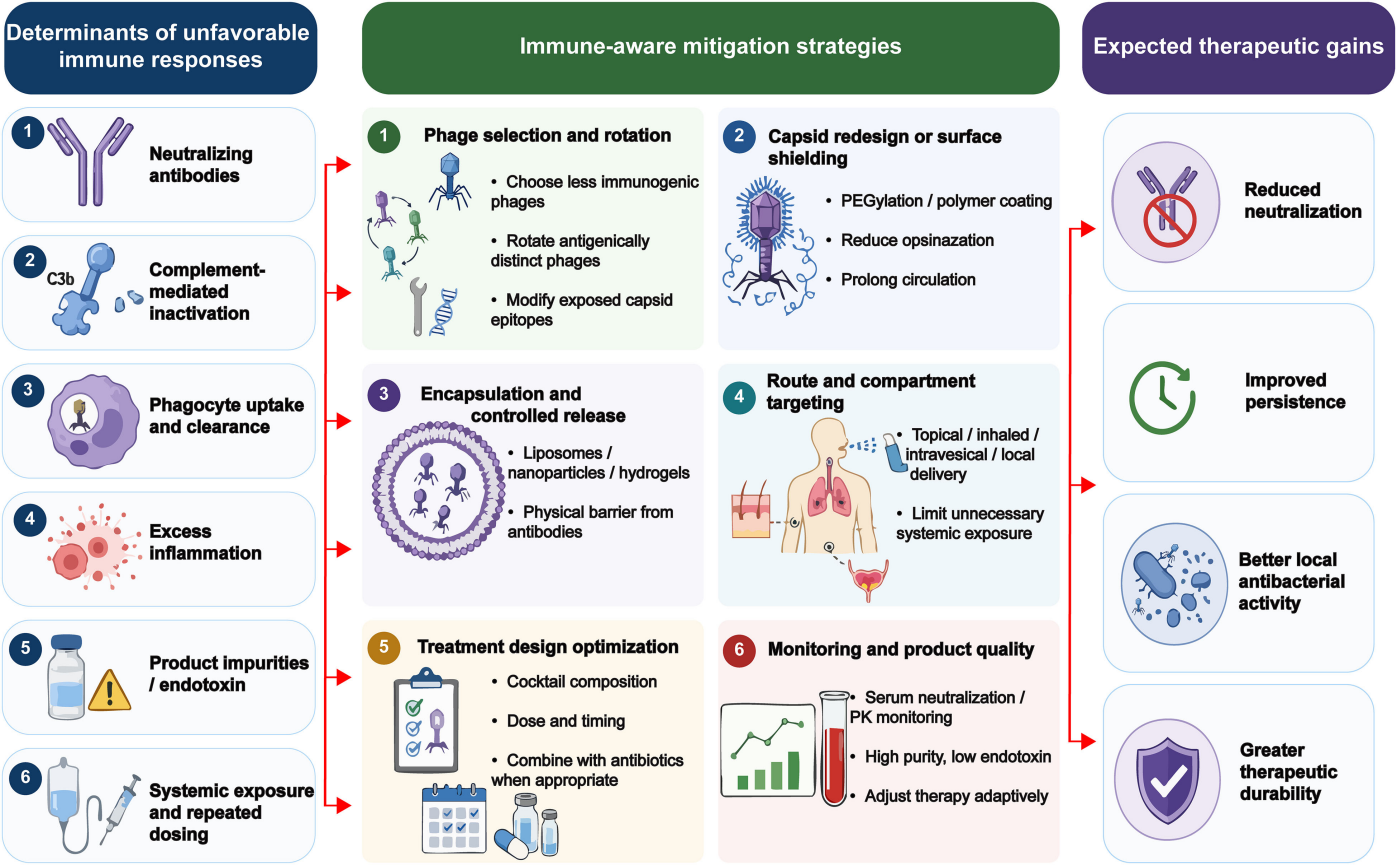
Overview of determinants of anti-phage immune responses and strategies to mitigate their impact on phage therapy. Pre-existing and treatment-induced immunity, complement activation, phagocytic clearance, inflammatory responses, impurities, and repeated exposure may reduce phage persistence and efficacy. Mitigation strategies include phage selection and rotation, capsid engineering or shielding, encapsulation, route optimization, treatment design, and immune monitoring, with the goal of improving phage persistence, reducing immune-mediated clearance, and enhancing therapeutic outcomes.

**Figure 2** summarizes the major determinants of unfavorable anti-phage immune responses, the principal mitigation strategies currently under investigation, and their anticipated therapeutic benefits.

## 8 Engineering, formulation and delivery strategies to manage immunity for phage therapy

Managing anti-phage immunity may be approached as a systems-design challenge in which phage particles and/or their biological programs are modified to reduce immune recognition when systemic exposure is required, while formulation and delivery strategies are optimized to confine phage exposure to the infection site and minimize the time window during which neutralizing antibodies can emerge (113). One of the most extensively studied “stealth” approach is capsid shielding (e.g., PEGylation or other polymer coatings) to sterically mask immunogenic surface proteins, reduce opsonization/clearance, and attenuate B−cell epitope accessibility; in a murine systemic MDR *E. coli* infection model, covalent 5 kDa PEGylation produced measurably lower anti-phage IgG than wild-type comparators while improving systemic persistence and exposure (AUC) and dampening infection-associated cytokines (114). However, these advantages are accompanied by important trade-offs, including reduced infective titer and adsorption, likely resulting from partial masking of receptor-binding interfaces, as well as the potential for anti-PEG antibodies in humans to diminish stealth performance and complicate repeated administration (115).

Beyond bulk polymer shielding, multiple protein/capsid engineering tactics are being pursued: mapping and modifying immunogenic capsid epitopes; altering net surface charge to reduce opsonin binding and prolong circulation (116); leveraging or emulating natural capsid glycosylation as an antibody-shield (117); and using genetic engineering (e.g., recombineering/CRISPR workflows) or selection to generate variants with reduced immune recognition or reduced immune activation (118). Conceptually, genome-level engineering could also mitigate innate immunostimulatory cues, but direct, clinically validated “deimmunized phage genomes” remain largely preclinical and will face high CMC/regulatory scrutiny (19).

On the formulation/delivery side, important immunity-management strategies include compartmentalization and controlled release: encapsulation in liposomes/nanoparticles and embedding in hydrogels can physically separate phage from neutralizing factors and provide controlled release at the target site while route selection (topical, intra-cavitary, intra-articular, inhaled/nebulized) aims to maximize local multiplicity-of-infection and reduce systemic antigen exposure versus IV (19). Human immunomonitoring data indicate why timing and route matter: phage therapy can elicit phage-specific CD4^+^ T-cell responses (including cTfh activity) alongside rising IgG and serum neutralization, with neutralizing activity first detectable around ~day 21 for a primary exposure and faster recall kinetics upon re-exposure. Yet, clinical benefit can still occur if phage dosing/availability remains sufficient (101). Conversely, other human experience shows that robust IgM/IgG neutralization can correlate with loss of therapeutic effect and discontinuation of IV phage in prolonged treatment, highlighting neutralization as a true, not merely theoretical, failure mode (80). Preclinical *in vivo* data further emphasize that phage choice matters: after repeat exposure in mice, neutralizing antibodies can accelerate tissue clearance and reduce efficacy on reuse, with stronger neutralization against certain phage families within a cocktail (e.g., some myophages > some siphophages) and comparatively modest T-cell responses at memory timepoints. These findings suggest that strategies such as phage rotation may be beneficial to antigenically distinct virions, minimizing systemic reuse of the same phage, and pre-treatment immunogenicity screening where feasible (28). Across strategies, the key trade-offs are predictable: shielding/encapsulation can reduce infectivity (slower adsorption, altered effective tropism), may change biodistribution/off-target interactions, and increases manufacturing characterization and regulatory complexity (especially for engineered genomes or coated/encapsulated products); immunomodulator co-administration or transient immunosuppression is conceptually plausible but carries obvious safety risks and, once memory B cells form, may offer diminishing returns relative to simpler route/formulation redesign (19).

In parallel with genetic and chemical engineering of phages, major advances in delivery and formulation are reshaping how phage therapy is implemented, with growing recognition that route of administration and formulation strongly determine how the immune system perceives, neutralizes, and clears therapeutic phages (86). Because phages encounter fundamentally different immune environments depending on where they enter the body, delivery strategy becomes a primary determinant of pharmacokinetics, immunogenicity, and therapeutic success. Mucosal exposure often operates under a baseline state of immune tolerance, and oral phage delivery has repeatedly been associated with relatively low systemic immune activation compared with systemic routes, even though repeated dosing can still generate measurable humoral responses over time (86). By contrast, systemic exposure places phages immediately into complement- and opsonin-rich environments that can reduce functional activity and accelerate clearance, motivating serum-aware selection and development strategies, including route choices that minimize unnecessary systemic exposure when local delivery is feasible (19). For pulmonary infections, inhalation offers direct access to infected airways and can achieve high local concentrations, but airway delivery also positions phages in close contact with alveolar macrophages and other innate sentinels, making immunophage synergy and local innate context central to outcomes (81). These observations support consideration of immune-aware delivery strategies: prioritize local administration when feasible and minimize unnecessary systemic exposure (86). At the mechanistic level, route and formulation also intersect with innate sensing programs: phage DNA can elicit antiviral-like transcriptional signatures through endosomal pathways such as TLR9 while canonical cytosolic antiviral execution may be muted, highlighting the importance of context in determining whether immune recognition translates into clinically meaningful inflammation (68).

To further reduce immune recognition and improve biodistribution, a major formulation focus has been to shield phage particles using encapsulation and controlled-release carriers, with the goal of limiting premature immune detection and preserving functional infectivity *in vivo*. This logic is supported by the broader observation that innate barriers can restrict effective exposure, and that immune memory and neutralization can diminish efficacy upon reuse. This makes strategies that prolong local residence or reduce systemic antigen exposure particularly attractive (28). Encapsulation platforms (e.g., liposomal or biomaterial-based carriers) may help address some of these bottlenecks by masking phage surfaces from opsonins and antibodies and enabling delayed release at infected sites, thereby sustaining local concentrations without repeated systemic dosing that could accelerate adaptive neutralization (119). Key design variables include carrier material, release kinetics, stability during nebulization/oral transit, and preservation of adsorption/infectivity at the target site (81). Finally, immune-aware delivery must be paired with rigorous product quality because local or systemic inflammatory risk is strongly shaped by impurities and consistency; this is reflected in translational emphasis on standardized characterization, purity/impurity control, and manufacturing robustness for phage therapy medicinal products (49).

## 9 Summary

Phage therapy represents a fundamentally different antibacterial modality in which clinical efficacy emerges from the coupled dynamics of phage amplification at the infection site, bacterial susceptibility, and host immune control. The evidence synthesized in this review shows that phages are not immunologically inert: they are detected, cleared, and sometimes functionally constrained by innate and adaptive mechanisms that place quantitative limits on persistence, especially during systemic delivery and repeated dosing, while simultaneously providing opportunities for synergistic bacterial clearance when host effectors and phage activity align. This duality helps explain why phage therapy can fail in some settings yet remain effective in others even when anti-phage antibodies are detectable, reflecting strong context dependence shaped by phage structure, formulation, route of administration, tissue compartmentalization, infection burden, and host immune state. Clinically, these insights require a shift from viewing phage therapy as a purely microbiological intervention to treating it as an immune-modulated biologic therapy, where phage selection and cocktail design incorporate not only host range but also immunological compatibility (including the risk of rapid cross-reactive neutralization), and where delivery route and formulation are chosen to balance local exposure against systemic immune elimination. Most importantly, the field is moving toward precision, immuno-informed phage therapy in which immune monitoring guides adaptive decisions such as phage rotation, dosing adjustments, and formulation changes, transforming phage therapy from empirical use into a rational, dynamically managed component of modern antimicrobial medicine.
